# Rapid epitaxy-free graphene synthesis on silicidated polycrystalline platinum

**DOI:** 10.1038/ncomms8536

**Published:** 2015-07-15

**Authors:** Vitaliy Babenko, Adrian T. Murdock, Antal A. Koós, Jude Britton, Alison Crossley, Philip Holdway, Jonathan Moffat, Jian Huang, Jack A. Alexander-Webber, Robin J. Nicholas, Nicole Grobert

**Affiliations:** 1 Department of Materials, University of Oxford, Oxford OX1 3PH, UK; 2 Oxford Instruments Asylum Research, High Wycombe HP12 3SE, UK; 3 Department of Physics, University of Oxford, Oxford OX1 3PU, UK; 4 Present address: Department of Nanostructures, Research Institute for Technical Physics & Materials Science, Budapest PO Box 49, Hungary

## Abstract

Large-area synthesis of high-quality graphene by chemical vapour deposition on metallic substrates requires polishing or substrate grain enlargement followed by a lengthy growth period. Here we demonstrate a novel substrate processing method for facile synthesis of mm-sized, single-crystal graphene by coating polycrystalline platinum foils with a silicon-containing film. The film reacts with platinum on heating, resulting in the formation of a liquid platinum silicide layer that screens the platinum lattice and fills topographic defects. This reduces the dependence on the surface properties of the catalytic substrate, improving the crystallinity, uniformity and size of graphene domains. At elevated temperatures growth rates of more than an order of magnitude higher (120 μm min^−1^) than typically reported are achieved, allowing savings in costs for consumable materials, energy and time. This generic technique paves the way for using a whole new range of eutectic substrates for the large-area synthesis of 2D materials.

## Abstract (web summary)

Innovative substrate engineering is necessary to improve the quality of CVD-synthesized graphene. Here the authors demonstrate *in situ* fabrication of an eutectic Pt-Si alloy that forms a wetting liquid surface on polycrystalline Pt foils, allowing millimetre-sized graphene crystals to grow in minutes.

## Graphical abstract

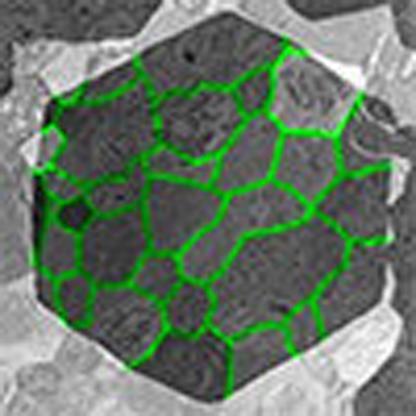

Graphene is a two-dimensional material that has great potential for use in a wide variety of applications such as transparent flexible electrodes, photonic and electronic devices, energy storage, sensors and coatings to name a few^1^. Exceptional electrical, optical and mechanical properties of the material greatly depend on the quality of the synthesized graphene that can inherently depend on the underlying substrate. Notably, the production of large area, single crystal graphene with a controlled number of layers is a challenge that has yet to be resolved.

Atmospheric pressure chemical vapour deposition (APCVD) has received much attention due to its simplicity, improved safety and lower cost over alternative procedures. When a solid substrate is used with APCVD, graphene growth is strongly influenced by the crystallographic lattice of the substrate, its defects, roughness and grain boundaries^2^,^3^,^4^,^5^. Millimetre-sized single-crystal graphene flakes have been synthesized recently on solid copper^6^ and platinum^7^. Time- and energy-consuming processes such as long annealing, mechanical, chemical and electrochemical polishing, re-solidification or their combinations were necessary to artificially increase the substrate grain size, eliminate the grain boundaries and decrease the roughness of the surfaces. Moreover, to produce large area, single crystal, monolayer graphene with CVD, the kinetics of the synthesis must be finely balanced. Typically this is achieved with low growth rates of a few μm per minute^7^,^8^. The synthesis time is thus lengthy (hours or days) and significant quantities of source materials are required. With unmodified commercially available polycrystalline Cu the achievable flake size is limited to only tens of micrometres^9^. It is also very difficult to achieve uniformity in the size and shape of graphene flakes within a sample on such substrates. Consequently, graphene films grown on solid polycrystalline metals often consist of small, irregular-sized domains with many high-angle domain boundaries, which degrade the quality of graphene^10^. CVD on liquid Cu gives aligned hexagonal graphene flakes of irregular size distribution of up to 120 μm or regular size distribution of 20–30 μm^11^. When these flakes coalesce they often have good crystallinity and low-angle grain boundaries. However, there is little control of the nucleation and the size of such flakes. The method is also difficult to upscale due to the liquidity or droplet formation.

In this investigation we combined the advantages of both approaches by using widely available, scalable, solid polycrystalline substrates in conjunction with a thin, liquid layer on the surface of these substrates. The layer is formed by coating polycrystalline Pt foils with a silicon-containing film, which forms a wetting liquid silicide surface *in situ* for graphene growth when heated, over the entire substrate surface. This significantly reduces the influence of the defects, roughness and the crystallographic orientations of the substrate on the quality of graphene. Substrates covered with the film exhibit increased growth rates of up to 120 μm min^−1^ at elevated temperatures, one of the fastest reported to date. While metal alloys^12^,^13^,^14^,^15^ and dielectric substrates^16^ have been shown, we believe this is the first eutectic system used for graphene synthesis by CVD. Interestingly, studies^17^ show that it is possible to oxidize intercalated silicides to achieve transfer-free graphene on an insulating surface, suggesting another potential benefit of the synthesis directly on metal silicides.

## Results

### Substrate preparation and large-area graphene synthesis

Pristine Pt for graphene synthesis was used as-received from the manufacturer followed by cleaning in acetone and deionized water. These substrates were not reused to avoid ongoing accumulation of Si-impurities. To form the silicidated Pt substrates, a SiO_2_ film was deposited on pristine Pt with a tetraethyl orthosilicate (TEOS) precursor in a CVD-based process, where TEOS was introduced by an Ar carrier gas (Methods). Other techniques such as evaporation and sol-gel deposition of Si/SiO/SiO_2_ were also investigated and showed similar trends after the optimisation of the procedure. CVD of TEOS is fast, cheap and can be performed in a single CVD system before synthesis. Upon annealing the SiO_2_ layer disappeared resulting in the formation of silicidated Pt (discussed later in the paper). To synthesize graphene, pristine Pt or the Pt/SiO_2_ stack was subjected to a CVD procedure that consisted of an annealing stage, synthesis stage and a cooling stage at atmospheric pressure (Methods). A compromise had to be made between the length of an experiment versus the size and quality of graphene flakes because the ratio of H_2_ to CH_4_ needed to be kept high^18^ to bias the CVD reaction towards single crystal, monolayer graphene growth. In our experiments we could differentiate three graphene growth scenarios in the vast parameter space available. For example, at a temperature of 1,070 °C with a hydrogen-to-methane ratio of 200:1, no growth could be observed after 30 min on silicidated Pt. A ratio of 160:1 at the same conditions resulted in the slow formation of high-quality monolayer graphene while rapid formation of multilayer graphene occurred at a H_2_:CH_4_ ratio of 100:1. The ratio of H_2_ to CH_4_ on silicidated Pt compared with pristine Pt had to be increased by as much as 50% (1,150 °C) to reduce the carbon deposition and achieve flakes of comparable size to those on pristine Pt. The comparison of the synthesis parameters on pristine and silicidated Pt is presented in Supplementary Fig. 1. This meant that the quality of graphene flakes improved without sacrificing the short experimental time. The difference in the kinetics for graphene nucleation and growth on pristine and silicidated Pt may be the key to explain this observation. Studies show that the bond strength of Si-C (∼318 kJ mol^−1^ (ref. 19)) is higher than that of Pt-C (∼225 kJ mol^−1^ (ref. 20)), but still lower than the graphene bond (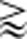
400 kJ mol^−1^ (ref. 21)) presenting a ‘stickier' silicide surface. This suggests that the associated dwell time of carbon atoms on the silicidated surface could be prolonged, leading to the observed increase in the growth rate.

**Figure 1:**
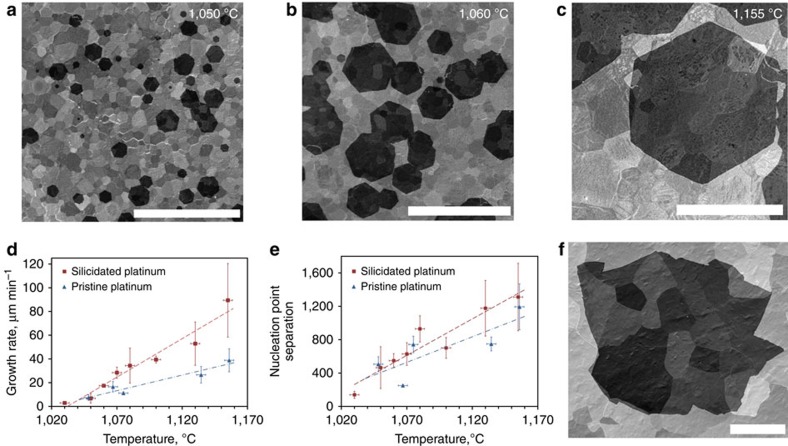
Graphene synthesis with CVD. (**a**–**c**) Representative SEM images of graphene on silicidated Pt for different temperatures: 1,050, 1,060 and 1,155 °C. (**d**,**e**) Graphene growth rate and its average nucleation point separation as functions of temperature. The vertical error bars are the approximate s.d. values calculated either from different experiments or from one experiment, whichever was larger. The trend lines are guides to the eye. (**f**) An SEM image of monolayer graphene on pristine Pt, with many reflex angles and varying edge morphology on different grains indicating polycrystallinity and strong dependence on the crystallographic orientation of Pt. Scale bars (**a**–**c**) 1 mm, (**f**) 100 μm.

Interestingly, the high melting point of Pt allowed us to probe unexplored temperatures above 1,100 °C in CVD, at which single crystal graphene flakes of up to 1.8 mm were obtained in 15 min on silicidated Pt. For comparison, the window of useful H_2_ to CH_4_ ratios on Cu is usually such that experiments often need to be conducted for hours or days^8^ to grow large-area, single crystal, monolayer graphene. Figure 1a–c depicts the improvement in the overall size of the flakes on the mm-scale that can be obtained with higher temperature on silicidated Pt. While graphene on pristine Pt showed similar trends, only smaller, polycrystalline flakes of irregular shape were observed throughout the CVD parameter space (Fig. 1f). The size of graphene flakes can be approximately determined by multiplying the growth rate by the synthesis time with the nucleation point separation as the maximum achievable graphene average domain size. The dependence of the growth rate as a function of temperature for silicidated and pristine Pt substrates is shown in Fig. 1d. As previously reported for Cu the nucleation density of the domains also decreased with higher temperatures^22^. However, on Pt the flake size was approximately an order of magnitude larger for the same temperature and time. The domain size of hexagonal or irregularly shaped graphene grown on Cu, Pt and silicidated Pt is related as follows Cu (1,050 °C)<Pt (1,050 °C)<Pt (1,150 °C)<silicidated Pt (1,150 °C). The difference in the nucleation point separation between pristine Pt and silicidated Pt is not as significant as the difference in the growth rate, yet it is still noticeable. The nucleation point separation (S), related to the nucleation density as π^−1^(S/2)^−2^, is also shown in Fig. 1e.

### Graphene characterization and electronic properties

A number of techniques were used to extensively assess the properties of the synthesized graphene, such as size, shape, crystallinity, number of layers and its electronics properties. Figure 1c,f shows two characteristic scanning electron microscope (SEM) images of graphene flakes on silicidated Pt and on pristine Pt. The latter had many reflex angles that indicated numerous grain boundaries^7^ (Supplementary Fig. 2), and the edge morphology often varied from one Pt grain to another, similar to what has been reported by Sun *et al*.^23^ and Ping & Fuhrer^24^. In contrast, the hexagonal graphene flakes of millimetre size grown on silicidated Pt were observed to cross sometimes hundreds of the Pt grains and their boundaries without any effect. Monolayer graphene was easily achieved on pristine Pt and silicidated Pt, most likely due to easily achieved ‘balance regime' between surface catalysis and segregation^23^. However, the crystallinity and size varied markedly depending on the experimental conditions. Contrary to the previous study of graphene synthesis on pristine Pt by Gao *et al*.^7^, where Pt was reused thousands of times inside a fused silica tube, we did not observe any hexagonal graphene flakes on pristine Pt that was not reused. The silicide surface is conductive and not easily distinguishable under an SEM, which may explain why the presence of silicides (Supplementary Fig. 3) has not been noticed and their striking influence on graphene growth in CVD has not been studied or reported before. The possibility to reuse Pt in combination with short experimental times can reduce costs and ensure the development of high-quality graphene syntheses commercially. We confirmed that the silicidated Pt substrates were also inert in the electrolyte solution (NaOH) that was used for a non-destructive graphene transfer (Methods) and thus could be reused.

**Figure 2:**
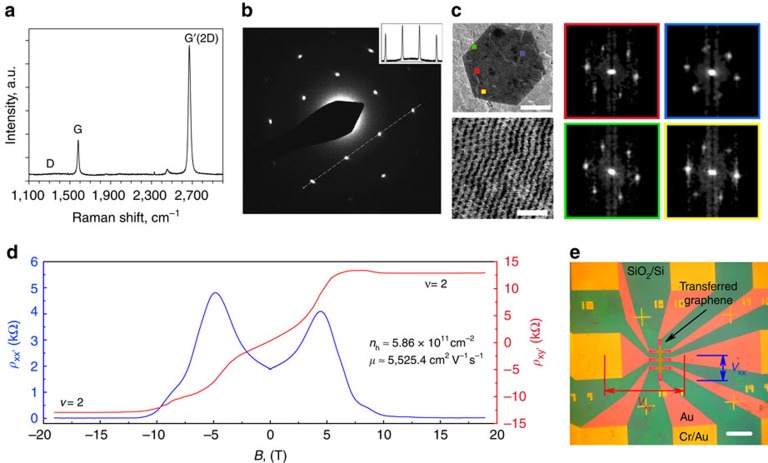
Graphene characterization and electronic properties. (**a**) Typical Raman spectra of graphene transferred to Si/SiO_2_ substrates from pristine or silicidated Pt, indicating high-quality monolayer graphene. (**b**) SAED of the synthesized graphene with a line profile (inset) indicating monolayer graphene. (**c**) Crystallinity analysis of graphene showing an SEM image of a hexagonal flake with marked areas of where lattice-resolved AFM scans were taken. An example of such a scan is shown underneath. These were performed directly on silicidated Pt. Two-dimensional Fourier transforms were taken (on the right) of the AFM scans; very small rotation of the FT patterns indicates good crystallinity over hundreds of micrometres. (**d**) *ρ*_*xx*_ and *ρ*_*xy*_ as functions of magnetic field. The carrier density and mobility shown were determined from the low-field Hall coefficient and the longitudinal resistance at 0 T. Also shown are the well-defined *ν*=2 quantum Hall states with quantized *ρ*_*xy*_ and vanishing *ρ*_*xx*_ at |*B*|*>*12 T. (**e**) The optical image of the actual device, where good contrast can be observed between the transferred graphene (light blue) and the SiO_2_/Si substrate (dark blue), and also between the Cr/Au (yellow) and Au-only (pink) contacts. On top of the Hall bar is the ma-N 2405 resist (brown). *V*_*xx*_ and *V*_*xy*_ are also labelled. Scale bars, (**c**) SEM flake: 200 μm, AFM scan: 1 nm; (**e**) 100 μm.

Graphene was transferred to Si/SiO_2_ substrates for Raman measurements (Fig. 2a). The D-peak was almost indistinguishable from the background indicating low defect concentration. The ratio of G′ to G peaks exceeded two over large areas, as expected for good quality monolayer graphene^25^, both for hexagonal and irregularly shaped flakes. Large-area graphene was transferred to plain transmission electron microscopy (TEM) grids (Au, Agar) without amorphous carbon support. TEM images and selected area electron diffraction patterns (SAED) were recorded. A line profile of the SAED pattern (Fig. 2b) provided additional supporting evidence for monolayer graphene^26^. The hexagonal shape of the flakes suggested good crystallinity, but to confirm this SAED patterns were recorded at points across large areas of a graphene flake on a TEM grid (Methods, Supplementary Fig. 2). In addition, lattice-resolution AFM scans of 6 nm × 6 nm were recorded on as-grown graphene on silicidated Pt and their Fourier transforms (FT) were taken (Fig. 2c). The roughness of the substrate after solidification was limiting the resolution of AFM images, but small-area scans were sufficiently flat and thus suitable for the FT analysis. These methods provided ways to measure the rotation of the lattice over distances of hundreds of micrometres. The measured values showed no significant changes in the rotation of the lattice and confirmed that the synthesized graphene was indeed a single crystal.

To assess the electronic properties of our CVD graphene a Hall-bar device (Fig. 2e) was fabricated from graphene transferred onto SiO_2_/Si substrates (methods) from silicidated Pt. The carrier density *n* and mobility *μ* of the device were determined from the low-field Hall measurements with *n*=(*e*·*∂ρ*_xy_/*∂B*)^−1^ and *μ*=(*enρ*_xx_)^−1^. Small p-type doping was observed in our device, which is also commonly seen in transferred graphene on SiO_2_/Si substrates and considered to be due to the adsorption of water molecules between the interfaces^27^,^28^. Figure 2d shows the *ρ*_xx_ and *ρ*_xy_ as functions of magnetic field from −19 to +19 T for a typical device. We observed a very high hole mobility of 5,525 cm^2^ V^−1^ s^−1^ at a hole density of 5.86 × 10^11^ cm^−2^. Recently, graphene has been shown to be an exceptional candidate for quantum Hall metrology^29^ exhibiting the highest breakdown current density of any material^30^. In our sample, at |*B*|*>*12 T, well-defined *ν*=2 quantum Hall states was clearly observed with *ρ*_xy_=*h/2e*^*2*^ and *ρ*_xx_=0, consistent with its carrier density 
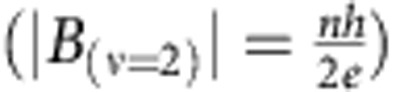
. These observations confirm the high quality of our monolayer graphene with good crystallinity, very low defects and impurities, and that graphene grown on silicidated platinum may be useful for quantum resistance metrology. The carrier mobilities obtained from the magnetotransport measurements are among the highest of reported values for CVD graphene^31^ on SiO_2_/Si substrates, and are comparable to those in epitaxial graphene grown on SiC (ref. 29). Using more sophisticated transfer methods or substrates with less charged impurities, such as *h*-BN^32^, further improvement of the carrier mobility is expected.

### Substrate characterization and silicidation mechanism

It is commonly known that Pt is a noble metal that is very inert; only hot aqua regia can react with it. Silicon dioxide (SiO_2_), fused silica or quartz may be described similarly due to the strong Si—O bond, which can be broken by hydrofluoric acid or some strong bases. However, when these compounds are in contact in a reducing H_2_ atmosphere at elevated temperatures silica may reduce to silicon, catalysed by Pt, and react with Pt to form various phases of eutectic Pt silicides as reported by numerous studies^33^,^34^,^35^,^36^. The two simplified reactions are as follows:


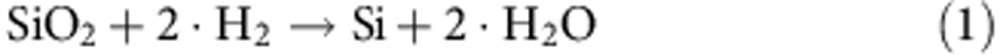



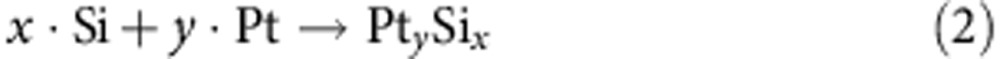


If pure silicon is deposited onto Pt, then the reaction proceeds much quicker and starts at lower temperatures. Other films, such as SiO, SiN also react in a similar manner. We found that a boron nitride holder was the most suitable for the Pt due to its stability in a reducing atmosphere and not-wetting by the silicide.

**Figure 3:**
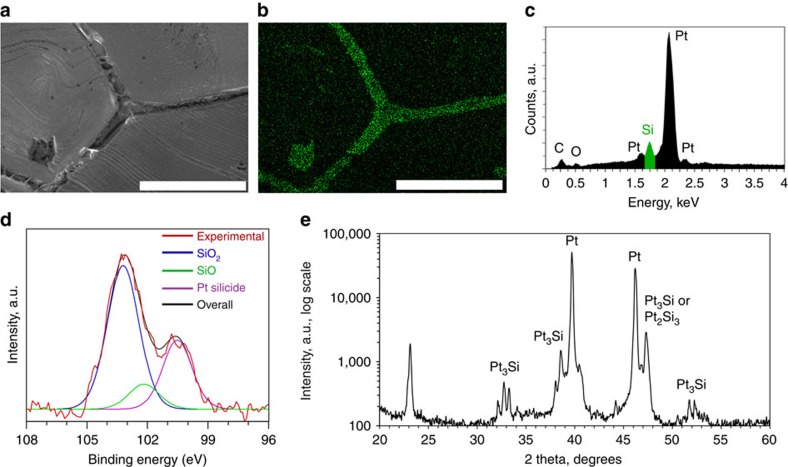
Characterization of the Pt/SiO_2_ substrate after annealing in hydrogen. (**a**) An SEM image of the annealed and cooled silicidated substrate with a filled Pt grain boundary. (**b**) EDX map of the Si peak, showing the valleys filled with a silicon-rich compound (green). The darker regions correspond to Pt grains with a much smaller Si-peak. (**c**) An EDX spectrum of the silicon-rich area from the grain boundary, where the Si peak is prominent. (**d**) XPS spectrum with fitted peaks, showing a matching silicide peak and silicon oxide peaks. (**e**) XRD spectrum of the substrate (on log scale), confirming the phase as Pt_3_Si (monoclinic when solid). Scale bars: (**a**,**b**) 30 μm.

**Figure 4:**
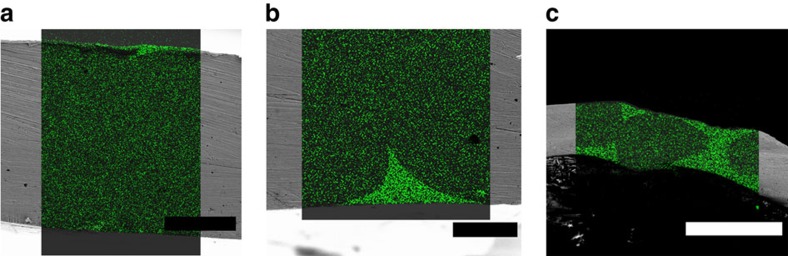
Bulk structure of the silicidated substrates. (**a**–**c**) Cross-sectional EDX maps of the silicon peak overlapped with SEM micrographs of the silicidated platinum samples. Low, medium and high silica thicknesses result in undetectable, medium and high silicide-phase presence. The silicon-containing regions and pristine Pt grains are completely indistinguishable in SEM mode. Restructuring and degradation of the foil can be observed for the samples with excess silica. Scale bars, (**a**) 10 μm, (**b**) 2 μm, (**c**) 50 μm.

Characterization of the annealed and cooled substrate before graphene synthesis was performed. The cooling was rapid (quenched) to obtain the closest possible condition to that during the synthesis. Figure 3a,b shows an SEM image of a grain boundary of the substrate and its corresponding energy-dispersive X-ray (EDX) spectral map of the silicon peak. The grain boundary and the small topographic defect have higher silicon content compared with the surroundings. The silicon peak is very prominent (Fig. 3c) in such valleys, but is typically much lower, but often detectable on the Pt grains. The presence of a variety of ‘frosted' features and striations in SEM also supported the formation of a thin silicide layer (Supplementary Fig. 3). X-ray photoelectron spectroscopy (XPS) revealed that Pt silicide covered the entire substrate (see peak at around 100.5 eV (ref. 37); Fig. 3d). Additional SiO/SiO_2_ peaks^38^ were also observed and are most likely formed when the silicide was exposed to air. Complimentary X-ray diffraction (XRD) analysis was performed to confirm the chemical composition, but also to identify the phase of the solidified silicide. For this, a thick SiO_2_ (∼3 μm) film was deposited on Pt to get the material in excess, resulting in the formation of a Pt_3_Si phase that matched well with the literature^39^ (Fig. 3e), but possibly other phases were present under different synthesis conditions. Cross-sectional EDX maps were recorded on Pt foils with a varying thickness of the silica layer to better understand the Pt/silica reaction and the chemical composition in the bulk. After the sample was annealed and quenched, regions with a thin silica layer (≲500 nm) did not show a detectable silicon peak in the EDX maps (Fig. 4a). Regions with a higher initial silica thickness (>500 nm) showed a high silicon signal (Fig. 4b) around the grain boundaries, suggesting that the reaction proceeded faster in those areas while progressing slowly into the bulk of the grains. Finally, excess silica (≳3,000 nm) resulted in the restructuring and degradation of the foil due to the formation of a two-phase composite (Fig. 4c), held together by the silicon-containing compound.

**Figure 5:**
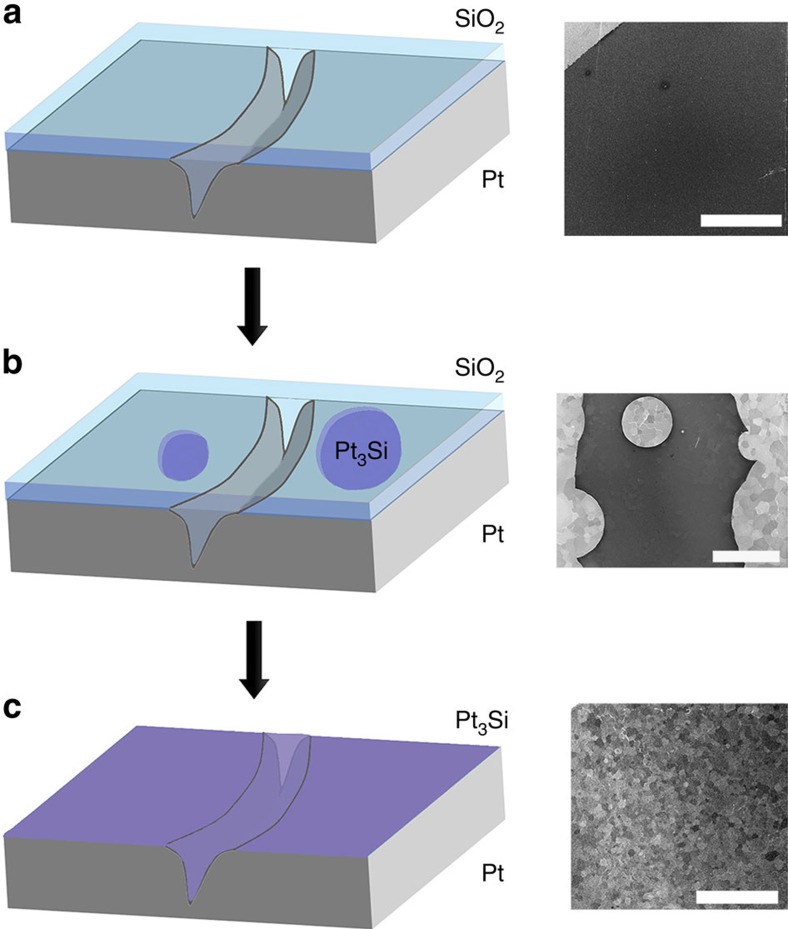
Stages of the silicide formation. (**a**) The substrate is coated with a SiO_2_ film. (**b**) Upon annealing a spontaneous reaction occurs resulting in circular holes in the film. (**c**) A thin liquid silicide layer covers the substrate and fills in any valleys or Pt grain boundaries. All the scale bars in the complimentary SEM micrographs are 1 mm.

On the basis of experimental observations of the SiO_2_ film before and after annealing we propose a simple mechanism of the liquid silicide formation that explains why the morphologies of graphene are so different on pristine and silicidated Pt. Most Pt silicide phases have reduced (eutectic) melting points^40^, especially in the form of a thin film. For example, the identified monoclinic Pt_3_Si phase melts at around 830 °C (ref. 40). Figure 5 shows a diagram of our proposed silicide formation mechanism and the corresponding SEM images of the Pt/SiO_2_ stack. The deposited silica layer is not conductive and appears dark under the electron beam. Upon heating, a spontaneous reaction occurs at a variety of points in the film that start to spread in a circular manner. We believe the circular holes in the silica film are filled with a thin layer of a conductive liquid, which when cooled appear similar to the pristine Pt but with a ‘frosted' surface (Fig. 5b,c, Supplementary Fig. 3). The silicide evaporates from the surface, diffuses into the bulk and locally smoothes any topographical defects, such as Pt grain boundaries, as inferred from the cross-sectional EDX maps in Fig. 4. It should be noted that the annealing time becomes an important parameter. Graphene synthesis can be started shortly after the SiO_2_ thin film completely reacts with Pt and the surface has a uniform chemical composition. We do not rule out chemical influences to the reaction mechanism by the silicidated Pt surface in addition to simple mechanical (liquid) effects. For example, the silicide route may produce silicon carbide as an intermediate state. Precise *in situ* studies would be needed to confirm the correct chemical mechanism. We also suggest that such eutectic materials may be suitable for use as liquid-surface substrates at low synthesis temperatures, bringing all the associated benefits.

### Graphene crystallinity control

**Figure 6:**
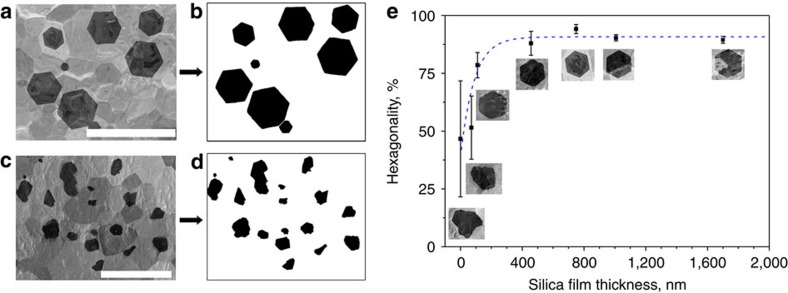
Influence of the silica layer thickness on the hexagonality and thus the crystallinity of the CVD graphene. (**a**,**b**) A sample with a high hexagonality and uniformity of graphene domains on silicidated Pt. A SEM micrograph is shown with a processed binary image of the resulting shape distribution. (**c**,**d**) A sample with low hexagonality and shape uniformity on pristine Pt. (**e**) With increased silica layer thickness the crystallinity and the shape uniformity increases. Error bars represent the distribution of shapes on the sample (Methods). The dashed line is a guide to the eye. Scale bars, (**a**) 400 μm, (**c**) 200 μm.

Good crystallinity is an important quality factor of CVD-grown graphene, as it offers superior electronic and tensile properties, chemical resistivity and impermeability^10^. The ability to control the crystallinity allows us to improve the material. A systematic investigation into the effect of the thickness of the deposited silica layer on the hexagonality of graphene flakes was conducted. We used a simple analysis technique to distinguish between different levels of hexagonality of the synthesized samples. Image processing was used to obtain a threshold to convert SEM micrographs into binary images of graphene domains (Fig. 6a–d). Each solid shape was then characterized individually with a computer script such that its pixel area and perimeter were measured to obtain a value relative to a perfect hexagon as detailed in the Methods section. The results are presented in Fig. 6e. The trend matches well with the expectation: the more silica is added, the thicker the liquid silicide layer is and thus the flakes grow more independently from the underlying solid substrate. The value of the hexagonality saturates at 800-nm-thick silica layer with a 91% similarity to a perfect hexagon. Another important quality indicator, the uniformity of the shapes, also decreases significantly from about 50% fluctuation at 0 nm to less than 5% at 800 nm, as shown by the error bars. Usable silica thicknesses are limited by the thickness of the Pt foil. If there is too much silica (>2 μm) the 25 μm Pt foil can start to degrade (Fig. 4c) or curl during the synthesis due to thermal stress caused because of the differences in the thermal expansion coefficients of Pt and silica. In addition, the silicide layer may undergo a phase change during cooling resulting in the formation of striations that may induce aligned cracks in graphene (Supplementary Fig. 3). A thin silica film is thus beneficial to avoid the formation of tall striations.

Although there is as yet no confirmed theory that explains the poor crystallinity of graphene on pristine Pt via CVD that we have observed, it is well known that the face-centred cubic (FCC) lattice of Pt has the largest lattice constant compared with the commonly used transition metals for graphene synthesis. On Cu(111) the lattice constant mismatch with graphene is 3.6%, whereas on Pt(111) this is significantly higher at 12.5%. This may result in incommensurable growth and the observed poor crystallinity.

## Discussion

In this study, we demonstrate a novel, low-cost substrate processing procedure to achieve rapid, efficient synthesis of millimetre-sized single crystal graphene. Silicidation of polycrystalline foils may be used as a replacement for expensive single-crystal substrates or as an alternative to polishing and grain enlargement. Large topographic defects, such as grain boundaries, are locally smoothed and the lattice of the grains is screened by a liquid surface, making silicidated polycrystalline foils superior to single crystals due to epitaxy-free synthesis. This ensures that graphene grows as a single crystal in its undistorted hexagonal shape. Silicidated polycrystalline Pt also exhibits low graphene flake nucleation density of up to 0.3 mm^−2^ and enhanced growth rates of up to 120 μm min^−1^ at elevated temperatures, allowing millimetre-sized flakes to grow easily in minutes. The silicide film formation on the substrates is an important parameter for graphene growth and may have beneficial effects if controlled. To our knowledge no current reports focusing on graphene synthesis via CVD consider the effects of the formation of the silicides, though commonly used reaction tubes are made of fused silica. It is likely that previous results were influenced by these silicides but were overlooked by other reports. In particular, we explicitly verify the dependence of the amount of silica on the crystallinity of graphene flakes on Pt. All of these beneficial effects may make the silicidation feasible commercially, especially if the procedure is extended to other metals. There is significant potential for further use of the method, such as a liquid surface for low temperature graphene synthesis or the possibility to oxidize the silicide layer to achieve transfer-free graphene. This approach is generally applicable and paves the way for a whole new field of eutectic substrates for the growth of superior quality 2D materials.

## Methods

### Substrate preparation

Pristine Pt substrates (25 μm, Goodfellow, 99.95% purity) were used as received from the supplier after cleaning in acetone and deionized water. To form a Pt/SiO_2_ stack, a film of SiO_2_ of specified thickness (Fig. 6e) was deposited onto pristine Pt via CVD with a tetraethyl orthosilicate (TEOS) precursor. For example, a SiO_2_ film of about 1 μm thickness can be deposited on pristine Pt with CVD by passing Ar gas (350 sccm) through a TEOS precursor (in a ‘bubbler') that is carried to a furnace with the substrates heated to 700 °C for 30 min. Other methods, such as Si, SiO, SiO_2_ evaporation, sputter coating, sol-gel processes or ion implantation are also applicable.

### Graphene synthesis

Pristine Pt or the Pt/SiO_2_ stack was placed on a boron nitride holder inside a fused silica tube (28 mm inner diameter) at sufficient distance from the walls of the tube. The tube was then purged with Ar (99.999% purity) and shifted into a pre-heated furnace, followed by annealing in H_2_ (99.995%, 200 sccm) to allow Pt silicide formation and to stabilize the temperature of the furnace. The useful annealing time to form silicidated Pt strongly depended on the temperature and the silica thickness. For example, at 1,070 °C with 1-μm-thick silica layer, 30 min was suitable (typically used, also for higher temperatures). However, at 1,050 °C with a 1 μm silica film after 30 min, 10% of the SiO_2_ film still remained. In the experiments where reusing silicidated Pt was tested, we shortened the annealing time to 5 min to simply re-heat the solidified silicide. To synthesize graphene, a mixture of CH_4_ (99.5%) and H_2_ was introduced. The gas ratio and the duration of the synthesis depended greatly on the temperature and the silica layer thickness and are given in the Supplementary Fig. S1 for both pristine and silicidated Pt. For example, for a 1-μm-thick silica layer at 1,070 °C the flow rates were set to 4 sccm CH_4_ and 600 sccm H_2_ for 20 min, resulting in ∼0.5-mm-sized graphene flakes. Directly after this step, the tube was shifted from the furnace and purged with Ar to quench graphene growth.

### Transfer to Si/SiO_2_ substrates and TEM grids

The ‘bubbling transfer' method outlined in Gao *et al*.^7^ was employed. The sample was spin-coated (500 r.p.m.) with polymethylmethacrylate (4% by weight PMMA in chlorobenzene, 996,000 Mw, Sigma-Aldrich) and submerged into an electrolyte solution (1 M NaOH). A potential difference was applied to the electrodes (6 V, Pt/graphene/PMMA on the negative terminal) submerged into the solution. This resulted in bubbles forming between the PMMA and Pt, separating them. PMMA with a graphene layer was then rinsed in deionized water and lifted onto a Si/SiO_2_ wafer, followed by heating at 140 °C for 5 min to improve the adhesion of graphene to SiO_2_. The stack was then placed in acetone (99.99%, Sigma-Aldrich) for 6 h and after dried with a N_2_ gun. For the TEM transfers, the PMMA layer was burnt in air at 350 °C after the transfer to a gold TEM grid, as outlined in Huang *et al*.^41^

### Graphene characterization

Raman spectra and maps were recorded with a Horiba LabRAM Aramis microscope equipped with a 532 nm laser. Routine SEM characterization was done with a JEOL JSM-6500F microscope at 5 kV. TEM images and SAED patterns were recorded with a JEOL JEM-2010 microscope at 80 kV. AFM measurements were performed using a Cypher AFM (Asylum Research). The scan was done in contact mode by looking at the lateral motion due to changes in friction as the probe scanned over the surface. To eliminate the effects of the direction of probe movement and also any topographic effects the trace scan was subtracted from retrace for the lattice-resolved image in real space. 2D fast Fourier transforms were taken of the AFM scans, the absolute values of which were then calculated and plotted on log scale; the contrast of the final images was adjusted to reduce the background.

### Electronic properties

Eight-leg Hall bars (15 μm × 130 μm) were fabricated using e-beam lithography followed by oxygen plasma etching. A two-step ohmic contacting method^42^ was used with thermally evaporated Cr/Au outer contacts and Au-only inner contacts (Fig. 2e). The final devices were coated in ma-N 2405 photoresist as a dielectric layer and a corona discharge technique^43^ was employed to control the charge carrier density. Electrical measurements were carried out at 1.4 K in an Oxford Instruments 21 T superconducting magnet, where magnetic fields *B* were applied perpendicular to the sample surface. A small AC current with peak amplitude of 40 nA was applied in the longitudinal direction (*I*_*xx*_). The longitudinal voltage *V*_*xx*_ and the Hall voltage *V*_*xy*_ (Fig. 2e) were simultaneously measured using the standard lock-in technique. The longitudinal resistivity *ρ*_*xx*_ and the Hall resistivity *ρ*_*xy*_ were determined as (*W*/*L*)˙*V*_*xx*_*/I*_*xx*_ and *V*_*xy*_*/I*_*xx*_, respectively, where *L* and *W* are the length and the width of the device.

### Substrate characterization

The thickness of the SiO_2_ films was measured with a Nanometrics Nanospec AFT interferometer on co-deposited Si tiles. Some samples were also analysed with ellipsometry directly on a Beaglehole Instruments Picometer Ellipsometer. The XPS measurements were performed at base pressure of 5 × 10^−10^ torr on a VG nine channel CLAM4 electron energy analyser with a 250 W Al X-ray excitation. XRD analysis was performed on the Bruker D5000 diffractometer.

### Hexagonality analysis

MATLAB was used to find an automatic threshold value of SEM micrographs, which were then converted into binary images. The ratio of the perimeter (*P*) squared to area (*A*) was calculated for each geometric shape. This unit-less number is usually indicative of specific geometric shapes. For example, for a circle it is 4π≈12.57 (the lowest possible value), for an octagon it is ≈13, for a hexagon √192≈13.86, for a pentagon ≈15, for a square it is 16 and so on. Concave polygons have higher *P*^2^/*A* values. If the calculated ratio (*P*^2^/*A*) for a shape is X, then we define the hexagonality as ≈1−|*X*−√192|/√192 (or as a percentage, multiplied by 100%). This means that a shape with a value close to 100% is close to a perfect hexagon. The s.d. of the % hexagonality for a sample indicates how non-uniform the shape distribution is. It is understood that rounded convex or concave polygons may introduce an uncertainty in the measurement, since, for example, a rounded cube may have the ratio of the perimeter squared to area similar to a hexagon. However, we usually have either hexagonal shapes (silicidated Pt) or concave polygons (pristine Pt); thus, for a large number of graphene domains the approach characterizes the samples sufficiently.

## Additional information

**How to cite this article:** Babenko, V. *et al*. Rapid epitaxy-free graphene synthesis on silicidated polycrystalline platinum. *Nat. Commun.* 6:7536 doi: 10.1038/ncomms8536 (2015).

## Supplementary Material

Supplementary Information Supplementary Figures 1-3
