# Supplementary material for: Rapid epitaxy-free graphene synthesis on silicidated polycrystalline platinum
Source: Nat Commun. 2015 Jul 15;6:7536. doi: 10.1038/ncomms8536 (PMC4518308; doi:10.1038/ncomms8536)
Supplement: Supplementary Information — Supplementary Figures 1-3 [file ncomms8536-s1.pdf]

SUPPLEMENTARY INFORMATION

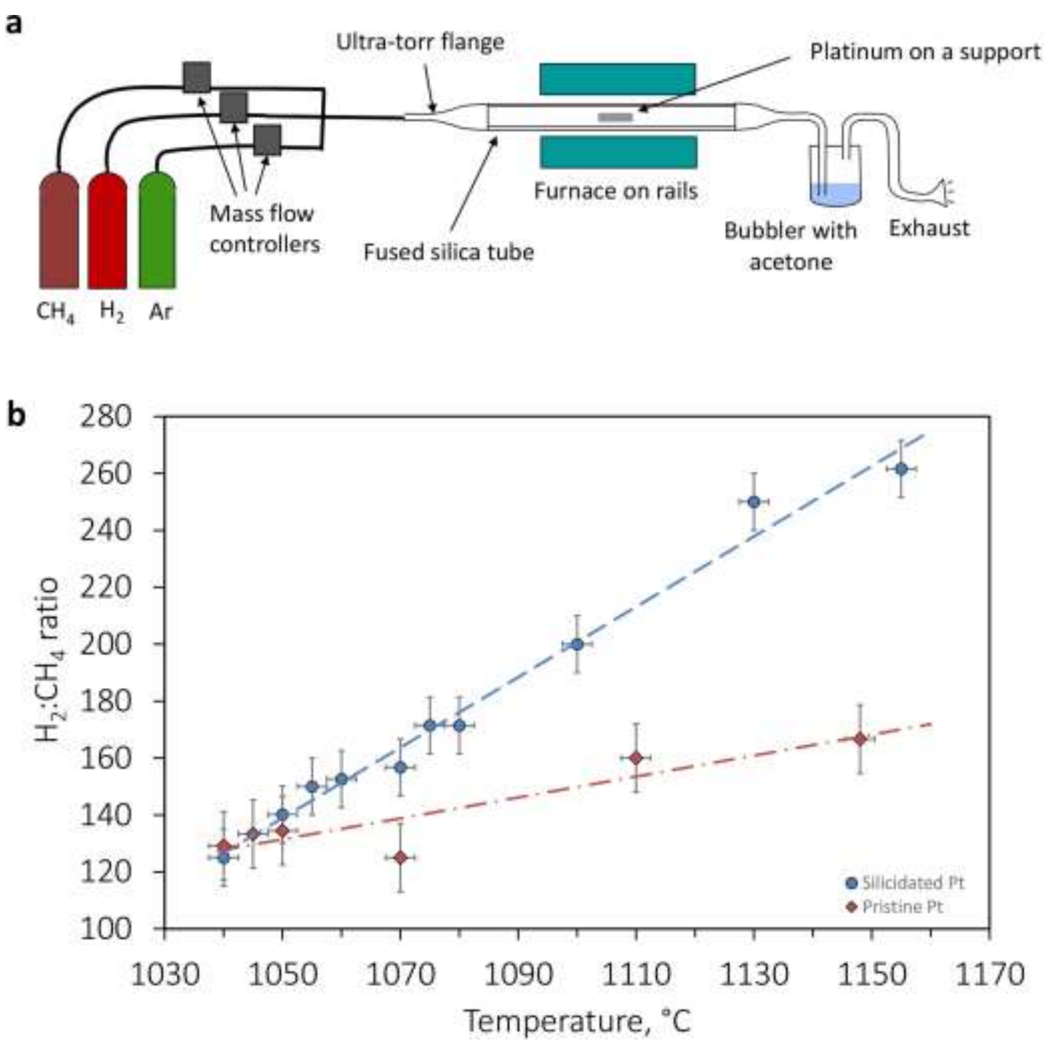

Supplementary Figure 1. Chemical vapour deposition synthesis procedure summary. a, A diagram of the APCVD system used for graphene synthesis consisting of a gas mixing manifold (CH4 99.5%, H2 99.995% and Ar 99.999% purity), a 28 mm inner diameter fused silica tube connected to the manifold and exhaust with ultra-torr fittings. To allow heating and rapid cooling of the samples the furnace (Elite TSH12/38/500) was shifted on a rail along the tube. **b,** The synthesis parameters for pristine and silicidated Pt that allow unconnected monolayer flakes to be synthesised. Silicidated Pt resulted in hexagonal flakes, while pristine Pt resulted in irregular-shaped flakes. Total flow rate and synthesis time were kept at around 600 sccm and 30 mins respectively.

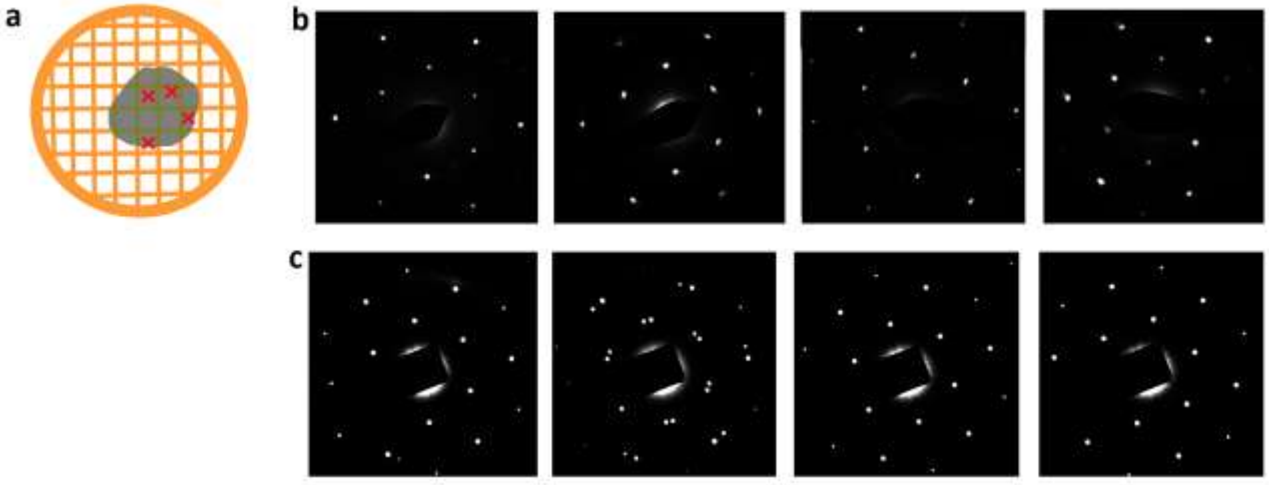

Supplementary Figure 2. Crystallinity characterisation of graphene with TEM. a, A schematic diagram of a graphene flake transferred to a TEM grid; the hexagonal shape is lost due to folding or damage to the edges that occurred during the transfer procedure. **b**, Corresponding SAED patters of various points of a graphene flake grown on silicidated platinum spanning a distance of over 200 µm, indicating a single crystal. **c**, SAED patters of an irregularly-shaped graphene flake grown on pristine platinum that consist of multiple single crystal domains (spanning tens of µm). Rotated SAED patterns of the domains comprising irregular-sized flakes are shown with a dual-hexagon pattern appearing near the domain boundaries.

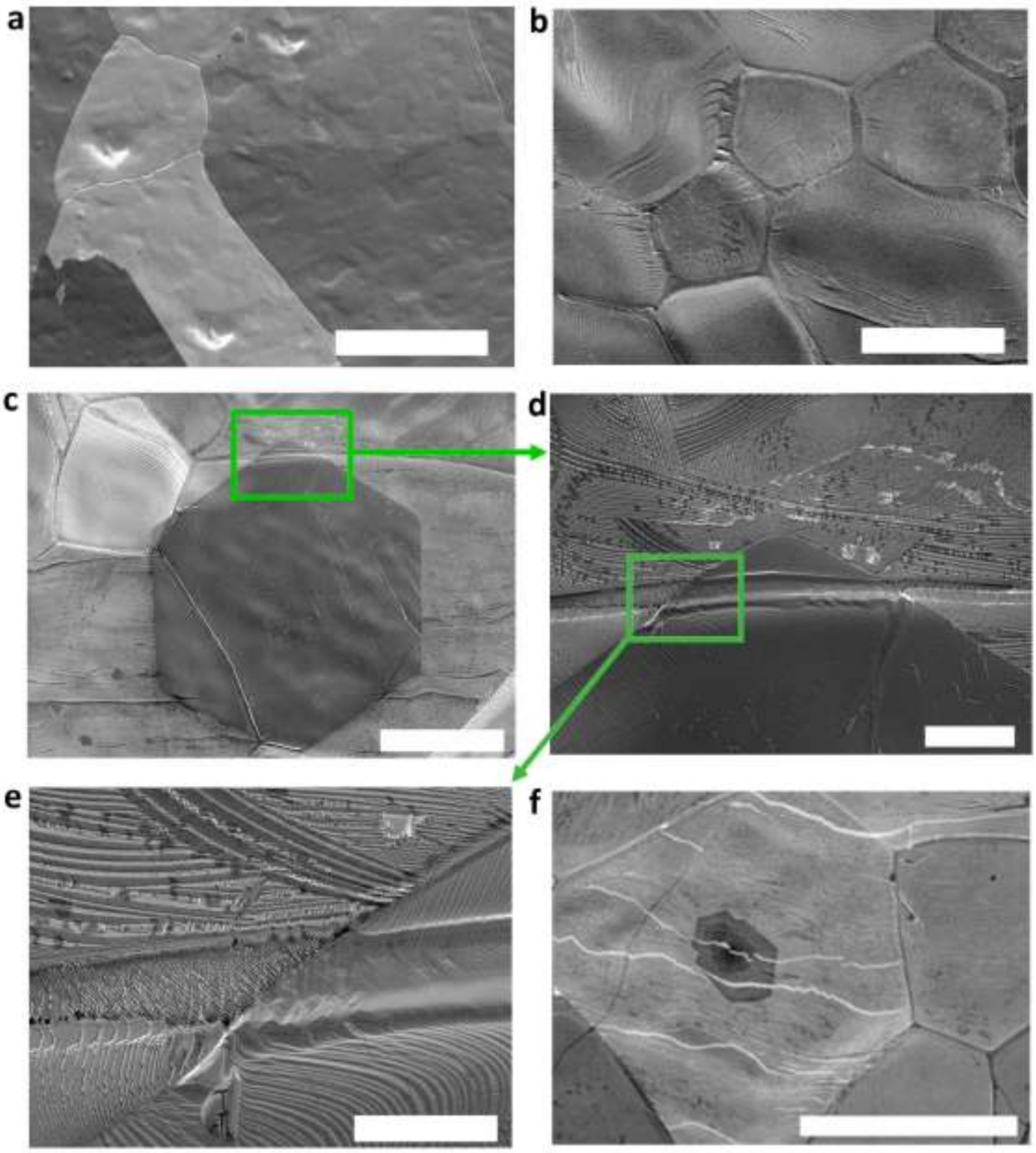

Supplementary Figure 3. Appearance of silicidated Pt and graphene in a scanning electron microscope. a, Pristine platinum from the manufacturer after annealing. **b**, Appearance of silicidated Pt in a SEM after cooling: striations, frosted features and thicker grain boundaries are noticeable, indicating liquid origins. From these features the presence of platinum silicide can often be deduced without additional chemical analysis. **c-d**, A small graphene flake extended over five grains. No change to the hexagonal shape is observed, confirming reduced dependence on the crystallographic orientation of the substrate and its grain boundaries. **e**, A high magnification micrograph of a graphene edge (right) crossing a grain boundary, showing high roughness of the re-crystallized silicide and a clear influence of graphene presence on the topography. **f**, Occasional, anisotropic crack formation in graphene was observed, perhaps due to strain induced by the silicide re-crystallisation or phase change. Scale bars are a-c) 50 µm, d) 10 µm, e) 5 µm, f) 100 µm.
